# The Myeloid-Epithelial-Reproductive Tyrosine Kinase (MERTK) rs4374383 Polymorphism Predicts Progression of Liver Fibrosis in Hepatitis C Virus-Infected Patients: A Longitudinal Study

**DOI:** 10.3390/jcm7120473

**Published:** 2018-11-23

**Authors:** María Ángeles Jiménez-Sousa, Ana Zaida Gómez-Moreno, Daniel Pineda-Tenor, Oscar Brochado-Kith, Juan José Sánchez-Ruano, Tomas Artaza-Varasa, Alicia Gómez-Sanz, Amanda Fernández-Rodríguez, Salvador Resino

**Affiliations:** 1 Unidad de Infección Viral e Inmunidad, Centro Nacional de Microbiología, Instituto de Salud Carlos III, Carretera Majadahonda-Pozuelo, Km 2.2, 28220 Majadahonda, 28220 Madrid, Spain; jimenezsousa@isciii.es (M.Á.J.-S.); obrochado@isciii.es (O.B.-K.); algomez@isciii.es (A.G.-S.); amandafr@isciii.es (A.F.-R.); 2 Servicio de Digestivo, Hospital Virgen de la Salud, 45004 Toledo, Spain; ana.zaidag@hotmail.com (A.Z.G.-M.); jjsanchezr@sescam.jccm.es (J.J.S.-R.); tdeartaza@gmail.com (T.A.-V.); 3 Unidad de Gestión Clínica de Laboratorio, Hospital de Antequera, 29200 Málaga, Spain; dpinedatenor@gmail.com

**Keywords:** liver stiffness, MERTK, chronic hepatitis C, cirrhosis, SNPs

## Abstract

Background: The myeloid-epithelial-reproductive tyrosine kinase (MERTK) is involved in hepatic steatosis, inflammation, and liver fibrosis. Here we evaluated the association between the *MERTK* rs4374383 single nucleotide polymorphism (SNP) and liver fibrosis progression in hepatitis C virus (HCV)-infected patients. Methods: We performed a retrospective study (repeated measures design) in 208 patients who had liver stiffness measurement (LSM), which was assessed using transient elastography. No patient had cirrhosis at baseline (LSM ≥ 12.5 kPa). Results: At baseline, 53.8% were male, the median age was 47.1 years, 13.5% reported a high intake of alcohol, 10.1% were prior injection drug users, 85.3% were infected with HCV genotype 1, and 22.6% had previously failed antiviral therapy (pegylated-interferon-alpha/ribavirin). During a median follow-up of 46.6 months, 26 patients developed cirrhosis. The rs4374383 G carriers had a higher risk of increasing LSM (adjusted arithmetic mean ratio (aAMR) = 1.14; *p* = 0.006) and a higher likelihood of having an increase in LSM greater than 5 kPa (ΔLSM ≥ 5 kPa) (adjusted odds ratio (aOR) = 2.37; *p* = 0.029), and greater than 7 kPa (ΔLSM ≥ 7 kPa) (aOR = 3.24; *p* = 0.032), after controlling for confounding. The SNP’s association with cirrhosis progression was close to statistical significance (aOR = 2.18; *p* = 0.070). Conclusions: *MERTK* rs4374383 A carriers had a lower risk of liver fibrosis progression than G carriers, supporting the hypothesis that this SNP seems to have a critical role in the pathogenesis of liver disease in HCV-infected patients.

## 1. Introduction

Chronic hepatitis C (CHC) leads to the development of hepatic fibrosis, cirrhosis, and end-stage liver disease [1], but its natural evolution is highly variable among hepatitis C virus (HCV)-infected patients since cirrhotic patients have a shorter survival time than those without cirrhosis [1]. Additionally, cirrhotic patients need specific and regular follow-ups with screening for hepatocellular carcinoma and esophageal varices [1]. Therefore, the staging of liver fibrosis is essential for the management and prognosis of patients with CHC [2], since early identification of patients at high risk for liver fibrosis progression is critical to ensure optimal preventive strategies [3]. The hepatic biopsy is the gold standard test to evaluate the severity of liver disease. However, this procedure has limitations and contraindications such as errors in samples, reading variability, hospitalization, cost, and delayed results, among others [4,5]. Given the drawbacks of liver biopsies, non-invasive tests for staging liver fibrosis have been developed during the last two decades, particularly the evaluation of liver stiffness measurement (LSM) using transient elastography, which can accurately predict the presence of liver fibrosis/cirrhosis in CHC patients [4,5]. Several cut-offs have been proposed to classify patients according to their fibrosis stage [5], but no general consensus has been reached for the different stages of fibrosis. One of the most commonly used in the last years has been the cut-offs proposed by Castera et al. [6] (7.1 kPa for *F* ≥ 2, 9.5 kPa for *F* ≥ 3, and 12.5 kPa for *F* = 4).

The pathogenesis of CHC is complex and is influenced by a variety of factors, many of which are still unknown. The most important among the known factors related to liver fibrosis progression in CHC patients are age at infection, sex, route of infection, HCV genotype, obesity, and single nucleotide polymorphisms (SNPs) [7,8]. An individual SNP may predispose an individual to the development of liver disease and may provide information on its pathogenesis. Despite this, the understanding of liver fibrosis progression and the host genetic factors involved in CHC have not been fully elucidated [3]. Furthermore, finding new genetic predictive factors for CHC progression may be essential for taking preventive measures [3].

The myeloid-epithelial-reproductive tyrosine kinase (MERTK), a member of the tumor-associated macrophage (TAM) family, is a tyrosine kinase receptor localized at the cytoplasmic membrane that has a crucial role in many physiological processes such as cell survival, migration, differentiation, and phagocytosis of apoptotic cells [9]. MERTK signaling attenuates innate immune responses through the modulation of proinflammatory cytokine secretion. Additionally, MERKT signaling promotes a Th2-like cytokines profile that favors wound healing and the resolution of inflammation [10]. Therefore, MERKT may be essential for regulating the liver inflammatory response against continual antigenic challenges, since it can prevent the over-activation of innate immunity [10]. In liver disease, TAM expression may be protective during acute liver injury, but TAM receptor signaling seems to be potentially deleterious in models of chronic liver disease [10]. MERTK is over-expressed in activated human and mouse hepatic stellate cells (HSCs), which promotes the progression of liver injury. Additionally, deficiency in growth arrest-specific 6 (GAS6), a ligand of MERTK, attenuates hepatic steatosis, inflammation, and liver fibrosis in mouse models [11,12,13]. The G allele of the *MERTK* rs4374383 SNP has been related to liver fibrosis severity in CHC [7,14]. The impact of this SNP on liver disease seems to be due to the loss-of-function of the rs4374383 G > A variant, a non-coding variant located within an intronic region of the *MERTK* gene, which could reduce HSC activation when the rs4374383 A-allele variant is present [13,14].

As far as we know, only three published reports have studied the association between the rs4374383 SNP and liver fibrosis in HCV infection, but all these studies were performed with a cross-sectional design [7,12,14], which may be a source of bias. In the current study, we evaluated the association between the *MERTK* rs4374383 SNP and the progression of liver fibrosis in HCV-infected patients via a longitudinal study.

## 2. Patients and Methods

### 2.1. Study Population

We carried out a retrospective cohort study (repeated measures design) in 208 HCV-infected patients who had values of LSM assessed using transient elastometry in Hospital Virgen de la Salud (Toledo, Spain) between 2008 and 2016. The study ran from the day of the first LSM value recorded to the last follow-up visit with LSM data, or the date of initiation of antiviral treatment for HCV in responder patients who cleared HCV infection. The administrative censoring date was 31 March 2016. 

This study was conducted according to the ethical standards given in the Declaration of Helsinki of 1975. The study was approved by the Institutional Review Board of the Instituto de Salud Carlos III, and all patients signed the consent.

The selection criteria of this study were: (1) detectable HCV RNA in plasma during all follow-ups, (2) a sample of DNA for genotyping, and (3) baseline LSM and final LSM in medical history with a least 12 months of difference. The exclusion criteria were: (1) cirrhosis (LSM ≥ 12.5 kPa) at baseline, and (2) hepatitis B virus infection or human immunodeficiency virus infection.

### 2.2. Clinical Data

Clinical and epidemiological data were obtained from medical records. We considered a high alcohol intake to be ≥60 g/day in men and >20 g/day in women [15]. The time since HCV diagnosis was calculated as the difference of time between HCV diagnosis and the first LSM (LSM1). The time of follow-up was the difference between the time between the last LSM (LSM2) and the first LSM (LSM1).

The management of the patients during follow-up was according to clinical guidelines [16,17], and HCV therapy could be used before or after the inclusion in the study. When patients were treated for HCV infection before starting the study, we only included non-responder patients. When a patient was treated for HCV infection after entering the study and achieved a sustained virological response (SVR), the follow-up was truncated to the time of beginning the HCV treatment.

### 2.3. HCV Assays

HCV infection was documented in all patients using enzyme-linked immunosorbent assays and polymerase chain reaction (PCR) tests. HCV genotype was determined by hybridization of biotin-labeled PCR products to oligonucleotide probes bound to nitrocellulose membrane strips (INNO-LiPA HCV II, Innogenetics, Ghent, Belgium). The plasma HCV RNA viral load was measured using real-time PCR (COBAS AmpliPrep/COBAS TaqMan HCV test), and results were reported in terms of international units per milliliter (IU/mL). The limit of detection was 15 IU/mL.

### 2.4. Genotyping of MERTK SNP

Total DNA was extracted from 200 μL of peripheral blood with QIAsymphony DNA Mini Kit (Qiagen, Hilden, Germany). The genotyping of DNA samples was performed at the Spanish National Genotyping Center (CeGen; http://www.cegen.org/) using Agena Bioscience’s MassARRAY platform (San Diego, CA, USA) and the iPLEX^®^ Gold assay design system [14]. The genotyping cluster plot is shown in Figure S1.

### 2.5. Liver Stiffness Measurement

LSM was assessed by transient elastography (FibroScan^®^, Echosens, Paris, France) and results were expressed in kilopascals (kPa) with a range of 2.5 kPa to 75 kPa [18]. Transient elastography was performed by a single trained hepatologist using a single machine. The measurements were made with at least four hours of fasting. Also, the measurements were considered to be reliable when the interquartile-range-to-median ratio for at least ten successful measurements was lower than 0.30. We used established cut-offs of LSM: <7.1 kPa (F0–F1—absence or mild fibrosis), 7.1–9.4 kPa (F2—significant fibrosis), 9.5–12.4 kPa (F3—advanced fibrosis), and ≥12.5 kPa (F4—cirrhosis) [6].

### 2.6. Outcome Variable

The main outcome was the increase in LSM values during follow-up (continuous variable). We evaluated the variation of LSM values between the last LSM (LSM2) and the first LSM (LSM1) as the ratio LSM2/LSM1 and the LSM increase (ΔLSM = LSM2−LSM1) by using a simple summary statistic approach to analyze the repeated measurements [19,20]. The use of the LSM2/LSM1 ratio provided the advantage that the resulting values could be normalized using a logarithmic transformation since LSM2/LSM1 never has negative values. Additionally, we used ΔLSM for evaluating long-term clinically relevant changes in LSM, which were changes of more than 5 kPa (ΔLSM ≥ 5 kPa), 7 kPa (ΔLSM ≥ 7 kPa), and 10 kPa (ΔLSM ≥ 10 kPa). The progression to cirrhosis (F4) may also have values of +1 (if F ≤ 3 (F0, F1, F2, or F3) changed to F4) or 0 (if F ≤ 3 did not change to F4).

### 2.7. Statistical Analysis

We condensed the information from repeat measurements to a single number per subject, which eliminates within-subject repeat measurements and allows a direct comparison of groups using standard tests of statistical hypotheses [19,20]. This strategy is very simple, provides valid results, and the results are easily understood.

The genetic association analysis was carried out according to additive, dominant, and recessive models of inheritance. The association of the *MERTK* rs4374383 SNP with the outcome variables was analyzed using generalized linear models (GLMs), with a gamma distribution (log-link) for continuous variables and a binomial distribution (logit-link) for dichotomous variables (ΔLSM ≥ 5 kPa, ΔLSM ≥ 7 kPa, and progression to F4). This test gives the differences between groups, the arithmetic mean ratio (AMR), and the odds ratio (OR), respectively. Each of the GLMs performed were adjusted by the main clinical and epidemiological characteristics: gender, age, time since HCV diagnosis, diabetes, high alcohol intake, HCV genotype, injection drug use, baseline LSM, HCV antiviral therapy before baseline and during follow-up (patients who failed therapy), and time of follow-up.

Statistical analyses were performed with the Statistical Package for the Social Sciences (SPSS) 21.0 software (IBM Corp., Chicago, IL, USA). All *p*-values were obtained using two-tailed tests. The statistical significance was defined as *p* < 0.05. 

## 3. Results

### 3.1. Baseline Characteristics of the Study Population

Table 1 shows the baseline characteristics of 208 HCV-infected patients without cirrhosis. Overall, 53.8% were male, the median age was 47.1 years, 13.5% reported a high intake of alcohol, 10.1% were prior injection drug users, 85.3% were infected with HCV genotype 1, and 22.6% had previously failed antiviral therapy (pegylated-interferon-alpha/ribavirin). There were no significant differences among patients with different *MERTK* rs4374383 genotypes at baseline. Eventually, 26 patients developed cirrhosis during a median follow-up time of 46.6 months.

### 3.2. Characteristics of the MERTK rs4374383 SNP

The rs4374383 SNP displayed <5% of missing values, had a minimum allele frequency >5%, and was in Hardy–Weinberg equilibrium (*p* > 0.05). Figure S2 shows genomic feature data surrounding the *MERTK* rs4374383 SNP from the UCSC Genome Browser (https://genome.ucsc.edu/).

### 3.3. MERTK rs4374383 SNP and Liver Fibrosis Progression

At baseline, we did not find any significant differences in LSM values and fibrosis stage according to *MERTK* rs4374383 genotypes (*p* > 0.05).

**Figure 1:**
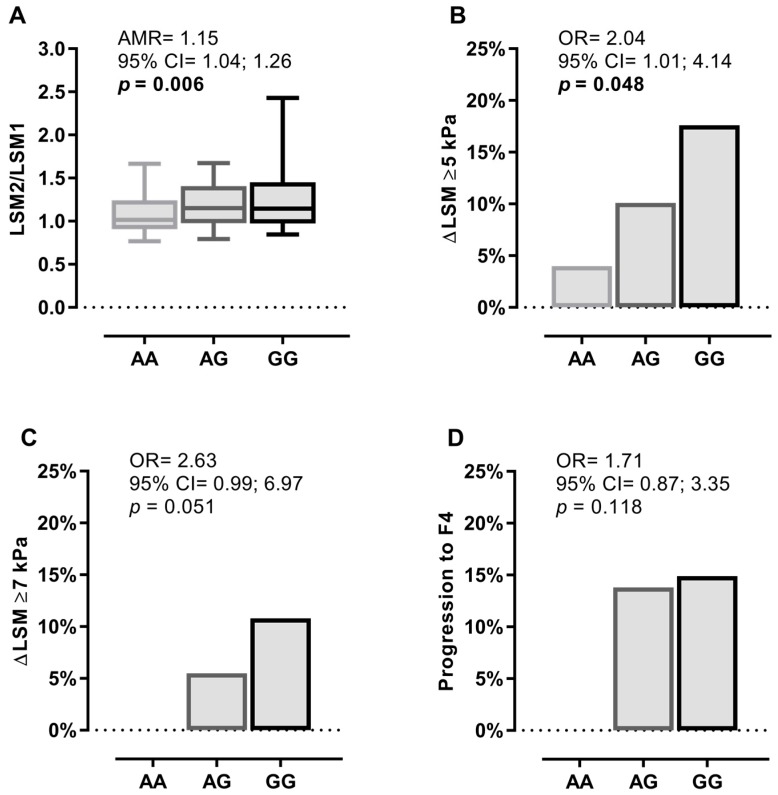
Summary of the frequencies and associations between the *MERTK* rs4374383 SNP and the change in LSM values and fibrosis stages in patients infected with HCV under an additive inheritance model. (**A**) shows the association of the *MERTK* rs4374383 SNP with LSM2/LSM1 ratio. (**B**) shows the association between the *MERTK* rs4374383 SNP and an increase in LSM greater than 5 kPa. (**C**) describes the association between the *MERTK* rs4374383 SNP and an increase in LSM greater than 7 kPa. (**D**) describes the association of the *MERTK* rs4374383 SNP and progression to cirrhosis (F4). Statistics: *p*-values were calculated using univariate regression models. The statistically significant differences are shown in bold. Abbreviations: MERTK, myeloid-epithelial-reproductive tyrosine kinase; *p*-value, level of significance; LSM, liver stiffness measure; Δ or delta, change in one variable (Δx (x2–x1)); LSM1, baseline LSM; LSM2, final LSM; F4, cirrhosis; kPa, kilopascal; AMR, arithmetic mean ratio; OR, odds ratio; 95% CI, 95% of confidence interval.

Figure 1 shows the univariate association between the rs4374383 SNP and clinical outcomes related to liver stiffness progression during follow-up under an additive model of inheritance, which was the one that best fit our data. Overall, we found a tendency towards greater liver fibrosis progression as the number of G alleles increased (additive effect). Thus, the rs4374383 G allele carriers (vs. A allele) had a higher risk of an increased LSM2/LSM1 ratio (AMR = 1.15; *p* = 0.006; Figure 1A) and having an increase in LSM greater than 5 kPa (OR = 2.04; *p* = 0.048; Figure 1B). No significant associations were found for an increase in LSM greater than 7 kPa and progression to cirrhosis (F4).

**Figure 2:**
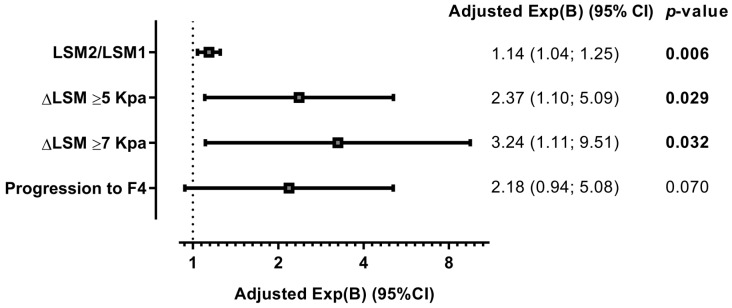
Summary of the adjusted association between the *MERTK* rs4374383 SNP and the change in LSM values and fibrosis stage in patients infected with HCV under an additive inheritance model. Statistics: *p*-values were calculated using multivariate regression models adjusted by the clinical and epidemiological characteristics (see statistical analysis section). The statistically significant differences are shown in bold. Abbreviations: MERTK, myeloid-epithelial-reproductive tyrosine kinase; *p*-value, level of significance; LSM, liver stiffness measure; Δ or delta, change in one variable (Δx (x2−x1)); LSM1, baseline LSM; LSM2, final LSM; F4, cirrhosis; kPa, kilopascal; Exp(B), exponentiation of the B coefficient, which was an arithmetic mean ratio (AMR) for continuous variable and an odds ratio (OR) for categorical variables; 95% CI, 95% of confidence interval.

Figure 2 shows that these tendencies were maintained in multivariate models adjusted by the principal clinical and epidemiological covariates, since the rs4374383 SNP was independently associated with greater LSM increases during follow-up under an additive model. Thus, the rs4374383 G allele carriers (vs. A allele) had a higher risk of an increased LSM2/LSM1 ratio (adjusted AMR (aAMR) = 1.14 (95% CI = 1.04; 1.25); *p* = 0.006); and having an increase in LSM greater than 5 kPa (ΔLSM ≥ 5 kPa) (aOR = 2.37 (95% CI = 1.10; 5.09); *p* = 0.029) and greater than 7 kPa (ΔLSM ≥ 7 kPa) (aOR = 3.24 (95% CI = 1.11; 9.51); *p* = 0.032). The association with progression to cirrhosis (F4) was relatively close to statistical significance (aOR = 2.18; *p* = 0.070).

We also found a significant association between the *MERTK* rs4374383 SNP and the LSM2/LSM1 ratio under dominant (AG/GG vs. AA) (aAMR = 1.28 (95% CI = 1.07; 1.55); *p* = 0.007) and recessive (GG vs. AA/AG) (aAMR = 1.13 (95% CI = 1.00; 1.28); *p* = 0.050) models of inheritance. Furthermore, HCV-infected patients with an AG/GG genotype had a higher frequency of progression to cirrhosis than patients with an AA genotype (14.2% vs. 0%; *p* = 0.044), but the logistic regression analysis could not be performed because one cell value was zero in the 2 × 2 contingency table. No association was found between the *MERTK* rs4374383 SNP and the dichotomous outcome variables (ΔLSM ≥ 5 kPa and ΔLSM ≥ 7 kPa) under dominant and recessive inheritance models.

Finally, we also tested the increase of 10 kPa (ΔLSM ≥ 10 kPa) with the three inheritance models (dominant, additive, and recessive), but we did not find any significant association either in univariate or multivariate analysis, possibly because the number of subjects with ΔLSM ≥ 10 kPa was very low (11 (5.3%)).

## 4. Discussion

In this longitudinal study, we found an association between the presence of the *MERTK* rs4374383 A allele and a reduced risk for liver disease progression in HCV-infected patients, whereas the G allele increased the risk. This association was found for the first time in a genome-wide association study (cross-sectional design) published by Patin et al. in the subset of transfused patients [14]. Next, two other articles, also with a cross-sectional design, studied such an association and obtained discrepant results [7,12]. On the one hand, Rüeger et al. showed that *MERTK* rs4374383 accelerated liver fibrosis progression by analyzing three additional independent cohorts and performing a meta-analysis with these data [7]. On the other hand, Kupcinskas et al. found similar distributions of alleles and genotypes between the control and liver fibrosis groups, describing a lack of association between *MERTK* rs4374383 and the odds of developing liver fibrosis or cirrhosis [12]. Additionally, Miyaaki et al. have also described a lack of association with liver fibrosis progression in liver biopsies after liver transplantation [21]. Regarding non-alcoholic fatty liver disease (NAFLD) among patients not infected by HCV, the *MERTK* rs4374383 A allele has also been associated with lower risk of liver fibrosis [13,22].

During hepatic fibrogenesis, activation of resident macrophages and further recruitment of inflammatory cells, including monocytes/macrophages, leads to the activation of HSCs [23]. MERTK has an essential role in the function of macrophages, including the clearance of apoptotic cells and cytokine secretion [24,25], and is therefore involved in the regulation of inflammatory responses and in hepatic fibrogenesis [10]. MERTK is upregulated in in vivo murine models of fibrosis and in activated mouse HSCs. Similarly, functional MERTK is expressed in activated human HSCs, and is involved in the induction of HSC migration, maintenance of fibrogenic cell survival, and overexpression of procollagen I [13]. Regarding the role of MERTK in the cross-talk between HSCs and inflammatory cells, it has been described that THP1 cells, a human leukemia monocytic cell line that can be differentiated into macrophages following stimulation by GAS6, induce an increase in the expression of profibrogenic factors in HSCs [23]. Therefore, all in all, MERTK plays an important role in the fibrotic process of liver diseases.

The association between the *MERTK* rs4374383 SNP and fibrosis progression seems to be mediated by the modulation of *MERTK* expression. In this sense, patients carrying the protective AA genotype had significantly lower hepatic *MERTK* expression, although the underlying mechanism is unknown [13]. The *MERTK* rs4374383 SNP is not located in a regulatory region, but a high number of SNPs are in high linkage disequilibrium (LD) (*r*^2^ > 0.8) with *MERTK* rs4374383 SNP. Thus, another SNP or SNPs in high LD could be causally responsible. This issue was investigated by Cavalli et al., who suggested that rs6726639A allele, in high LD with rs4374383 (*r*^2^ = 0.94 according to data from 1000 genomes), could promote the binding of interferon regulatory factor 1 (IRF1) to this region [26], and serve to activate or repress the expression of a high number of genes involved in the immune response [27]. The preferential binding of IRF1 to the A allele compared to the C allele would downregulate MERTK in patients carrying the A allele, protecting against liver fibrosis and hepatocarcinoma [26]. Functional studies would be needed to identify the causal SNP. However, due to the high LD between rs4374383 and rs6726639, both SNPs would provide similar information in a statistical analysis of genetic association like the one carried out in this study. Therefore, the two SNPs (rs4374383 and rs6726639) may be interchangeable for predicting liver fibrosis progression.

Our study has important advantages in design compared to previous articles that studied the association between *MERTK* rs4374383 SNPs and progression of liver fibrosis. All of them were cross-sectional studies. Patin et al. carried out a study with a classic case/control approach (F0–1 vs. F3–4, and F0 vs. F4). They also performed a survival analysis and investigated the role of the *MERTK* rs4374383 SNP on fibrosis progression rate (FPR) [14] by using, in both approaches, the estimated duration of HCV infection. However, it could lead to an inherent bias because of the estimates. Similarly, Rüeger et al. performed a cross-sectional study using the estimated FPR as an outcome [7], and Kupcinskas et al. performed a case/control study [12] to evaluate the effect of *MERTK* rs4374383 on the risk of liver fibrosis and cirrhosis. In contrast, the present study has a longitudinal design with repeated measures (the change of LSM was evaluated during the follow-up in each patient). Thus, we evaluated two LSM values from each subject over a period of at least 12 months. This approach offers a better ability to detect fibrosis changes over time with fewer subjects.

## 5. Limitations of Study

Several study limitations must be considered for the correct interpretation of the results. First, the study design is retrospective, thus bound to ascertainment and selection biases. Many patients may have undergone LSM measurements for a reason (e.g., sudden increases of liver enzymes), while others may have failed to undergo repeated testing and thus were excluded from the analysis. Second, the limited sample size of this study could limit the ability to find significance in some comparisons, such as the association between *MERTK* rs4374383 and the progression to cirrhosis. However, the longitudinal design with repeated measures improves the statistical power of this study considerably. In this respect, it should also be noted that the time between LSM1 and LSM2 (follow-up time) was different between patients, but all patients had at least 12 months of follow-up and 75% of patients had at least 28.7 months of time between LSM1 and LSM2 (median of follow-up: 46.6 (P25th: 28.7; P75th: 61.5) months). Besides, we included the follow-up time as an adjustment variable in the multivariate analyses. Third, we did not have data for some major variables, such as metabolic syndrome, obesity, and hepatic steatosis, since these variables were not routinely evaluated for all patients. Fourth, patients who did not respond to interferon therapy were included, since interferon treatment failure appears not to protect patients from the natural progression of fibrosis over time [28]. Fifthly, we used the change in LSM to define liver fibrosis progression, and this measurement may not be robust enough since there may be many factors that affect the LSM (e.g., antiviral therapy, change in body weight, etc.).

## 6. Conclusions

In conclusion, *MERTK* rs4374383 A carriers had a lower risk of liver fibrosis progression than G carriers, supporting the hypothesis that this SNP seems to possess a critical role in the pathogenesis of liver disease in HCV-infected patients. Moreover, further studies should be conducted to evaluate the impact of the *MERTK* rs4374383 SNP on liver fibrosis and the development of hepatocellular carcinoma after HCV elimination since patients with advanced fibrosis or cirrhosis are still at risk of disease progression [29].

## Figures and Tables

**Table 1:** Clinical and epidemiological characteristics of HCV-infected patients stratified by *MERTK* rs4374383 genotypes.

Characteristic	All Patients	MERTK rs4374383 SNP		
		AA	AG	GG
**No.**	208	25	109	74
**Male**	112 (53.8%)	15 (60%)	59 (54.1%)	38 (51.4%)
**Age (years)**	47.1(41.5; 57.6)	42.3 (38.2; 49.1)	47.6 (42.2; 59.5)	49.1 (42.7; 58.7)
**Time of HCV infection (years)**	8.2 (3.2; 13.2)	7.2 (3.9; 16.7)	9.8 (3.3; 13.3)	6.4 (2.7; 12.1)
**High alcohol intake**	28 (13.5%)	6 (24%)	13 (11.9%)	9 (12.1%)
**Prior injection drug use**	21 (10.1%)	6 (24%)	8 (7.3%)	7 (9.5%)
**HCV genotype (*n* = 204)**				
1	174 (85.3%)	17 (68%)	91 (86.7%)	66 (89.2%)
3	14 (6.9%)	4 (16%)	7 (6.5%)	3 (4.1%)
4	15 (7.4%)	4 (16%)	6 (5.7%)	5 (6.8%)
5	1 (0.5%)	0 (0%)	1 (1%)	0 (0%)
**Prior failed peg-IFN-α/RBV therapy**	47 (22.6%)	4 (16%)	30 (27.5%)	13 (17.6%)
**Baseline LSM (kPa)**	6.1 (5.2; 7.7)	6.4 (4.6; 7.4)	6.3 (5.1; 8.0)	6 (5.3; 6.8)
F0–F1 (<7.1 kPa)	149 (71.6%)	17 (68%)	72 (66.1%)	60 (81.1%)
F2 (7.1–9.4 kPa)	38 (18.3%)	6 (24%)	25 (22.9%)	7 (9.5%)
F3 (9.5–12.4 kPa)	21 (10.1%)	2 (8%)	12 (11%)	7 (9.5%)
**Follow-up time (months)**	46.6 (28.7; 61.5)	48.6 (30.1; 64.1)	47.9 (28.9; 60.6)	45.2 (25.5; 61.5)
**Final LSM (kPa)**	6.8 (5.5; 9.4)	6.3 (5.1; 8.7)	7.4 (5.9; 10.1)	6.8 (5.4; 8.9)
F0–F1 (<7.1 kPa)	110 (52.9%)	17 (68%)	53 (48.6%)	40 (54.1%)
F2 (7.1–9.4 kPa)	47 (22.6%)	3 (12%)	28 (25.7%)	16 (21.6%)
F3 (9.5–12.4 kPa)	25 (12%)	5 (20%)	13 (11.9%)	7 (9.5%)
F4 (≥12.5 kPa)	26 (12.5%)	0 (0%)	15 (13.8%)	11 (14.9%)

Values expressed as absolute numbers (%) and median (percentile 25; percentile 75). Abbreviations: HCV, hepatitis C virus; LSM, liver stiffness measure; kPa, kilopascal; peg-IFN-α/RBV, pegilated-interferon-alpha/ribavirin; MERTK, myeloid-epithelial-reproductive tyrosine kinase.

## Supplementary Materials

The following are available online at http://www.mdpi.com/2077-0383/7/12/473/s1, Figure S1: Genotyping cluster plot for MERTK rs4374383 polymorphism, Figure S2: Genomic features from data tracks in the UCSC Genome Browser (https://genome.ucsc.edu/) in the region around MERTK rs4374383 SNP.
