# Supplementary material for: The Myeloid-Epithelial-Reproductive Tyrosine Kinase (MERTK) rs4374383 Polymorphism Predicts Progression of Liver Fibrosis in Hepatitis C Virus-Infected Patients: A Longitudinal Study
Source: J Clin Med. 2018 Nov 23;7(12):473. doi: 10.3390/jcm7120473 (PMC6306820; doi:10.3390/jcm7120473)

Figure S1: Genotyping cluster plot for MERTK rs4374383 polymorphism.

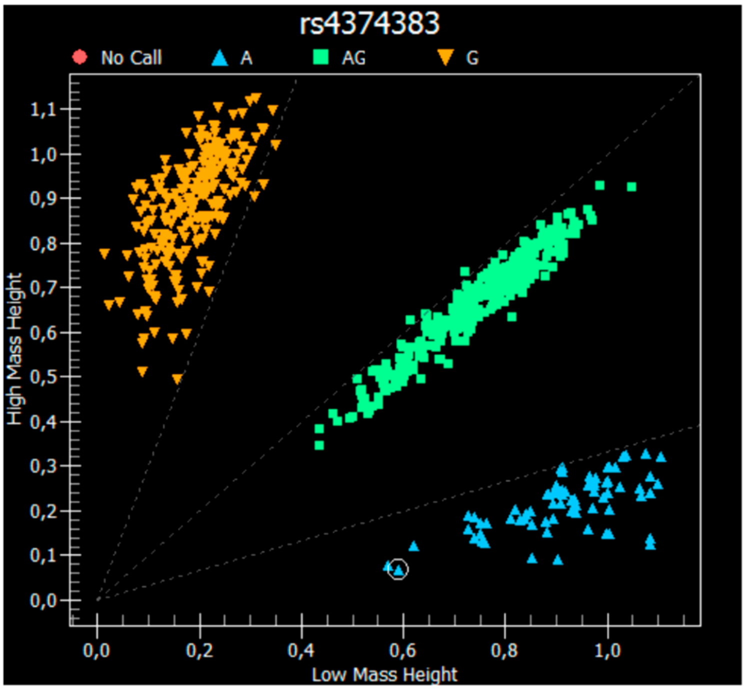

Figure S2: Genomic features from data tracks in the UCSC Genome Browser (https://genome.ucsc.edu/) in the region around *MERTK* rs4374383 SNP.

This figure presents genomic feature data obtained from the UCSC Genome Browser for the region of chr2:111,898,479-112,029,561 (GRCh38/hg38 assembly), which encompasses the *MERTK* gene. The position of *MERTK* rs4374383 SNP is highlighted by a vertical blue line. The following data tracks are shown in the figure: NCBI RefSeq genes, Gene Expression in different tissues from GTEx RNA-seq, CpG islands, histone modifications, DNase I Hypersensitivity Peak Clusters from ENCODE, Regulatory elements from Open Regulatory Annotation database (ORegAnno), Conservation by PhyloP, Single Nucleotide Polymorphisms found in >= 1% of samples and Repeating Elements by RepeatMasker.

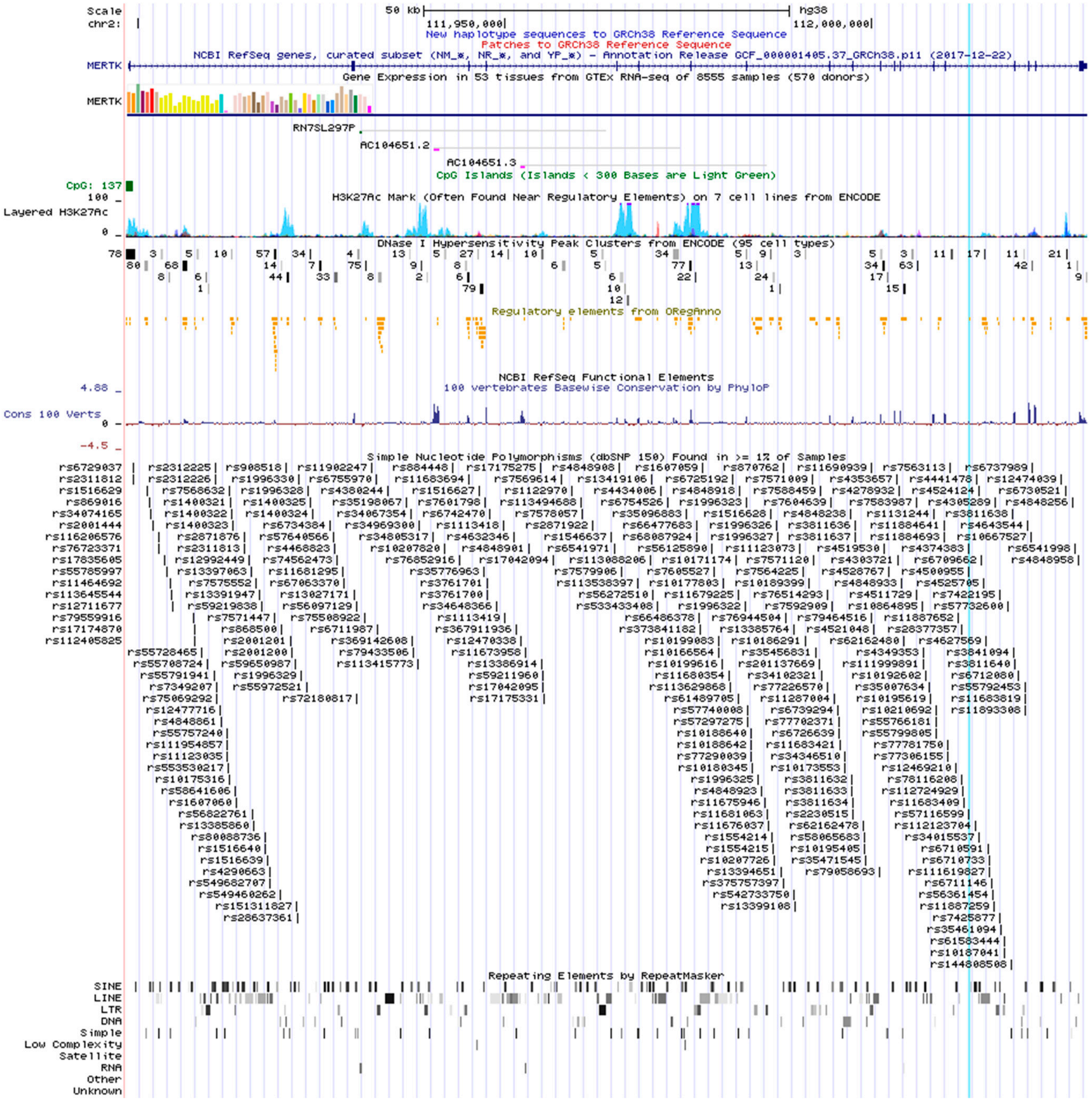

Supplement: Supplementary file 1 [file jcm-07-00473-s001.pdf]
